# Roles of Serum Calcium, Phosphorus, PTH and ALP on Mortality in Peritoneal Dialysis Patients: A Nationwide, Population-based Longitudinal Study Using TWRDS 2005–2012

**DOI:** 10.1038/s41598-017-00080-4

**Published:** 2017-02-24

**Authors:** Chung-Te Liu, Yen-Chung Lin, Yi-Chun Lin, Chih-Chin Kao, Hsi-Hsien Chen, Chih-Cheng Hsu, Mai-Szu Wu

**Affiliations:** 1 Division of Nephrology, Department of Internal Medicine, Taipei Medical University-Wanfang Hospital, Taipei, Taiwan; 2 Department of Internal Medicine, School of Medicine, College of Medicine, Taipei Medical University, Taipei, Taiwan; 3 Graduate Institute of Clinical Medicine, College of Medicine, Taipei Medical University, Taipei, Taiwan; 4 Division of Nephrology, Department of Internal Medicine, Taipei Medical University Hospital, Taipei, Taiwan; 5 Division of Endocrinology & Metabolism, Department of Medicine, Taipei Veterans General Hospital, Taipei, Taiwan; 6 Faculty of Medicine, National Yang-Ming University, Taipei, Taiwan; 7 Institute of Population Health Sciences, National Health Research Institutes, Zhunan, Taiwan

## Abstract

Biomarkers of chronic kidney disease-mineral and bone disorder (CKD-MBD) correlate with morbidity and mortality in dialysis patients. However, the comparative roles of each CKD-MBD biomarker remained undetermined on long-term peritoneal dialysis (PD) patients. This retrospective study, employing a population-based database, aimed to evaluate the performance and provide the best evidence of each biomarker of CKD-MBD as predictor of all-cause mortality. Throughout the 8-year study period, total 12,116 PD patients were included in this study. Cox proportional regression and Kaplan-Meier method were used for survival analysis. For Cox regression model, baseline measurements and time-varying covariates were used for analysis. In Cox regression model using time-dependent covariates, serum calcium level of ≧9.5 mg/dL was associated with increased mortality. For phosphorus, serum levels of either ≧6.5 mg/dL or <3.5 mg/dL were associated with increased mortality. For parathyroid hormone (PTH), higher serum levels were not associated increased mortality. For alkaline phosphatase (ALP), mortality increased at levels ≧100 IU/L. Our findings suggested that the detrimental effect of ALP on survival was more consistent, while serum calcium, phosphorus and PTH may have a less prominent effect on mortality. This study provided additional information for manipulating CKD-MBD biomarkers in PD patients.

## Introduction

In the last two decades, numerous studies have provided evidence that biochemical changes in chronic kidney disease-mineral and bone disorder (CKD-MBD) are associated with survival outcomes in dialysis patients. Observational studies have demonstrated that adverse cardiovascular outcomes and mortality in dialysis patients are associated with high levels of serum phosphorus^1–8^ and calcium^1–5^, parathyroid hormone (PTH) levels either higher^3–7^ or lower^9, 10^ than the target range, and elevated alkaline phosphatase (ALP) levels^11–13^. These findings have contributed significantly to the widely accepted Kidney Disease Improving Global Outcomes (KDIGO) clinical practice guidelines for CKD-MBD^14, 15^.

While the majority of studies focusing on CKD-MBD in dialysis patients involved haemodialysis (HD) patients, relatively few studies involving peritoneal dialysis (PD) patients have been published^16, 17^. Recently, several studies have revealed that an association between ALP level and mortality also exists in PD patients^18–20^. These studies form the basis for the current management of CKD-MBD in PD patients. Nonetheless, the amount of evidence supporting the management of CKD-MBD in PD patients remains relatively small compared to that for HD patients. Thus, a comprehensive, large-scaled study focusing on CKD-MBD in PD patients would be help to improve our understanding in this field.

In practice, adherence to every recommendation for CKD-MBD in a single dialysis patient is not always feasible. For example, the use of calcitriol to suppress the PTH level may result in an adverse elevation of serum calcium and phosphorus levels. In addition, use of a calcium-based phosphate binder may inappropriately increase calcium load and serum calcium level. As such, providing optimal treatment for each dialysis patient is difficult, particularly as the relative role of each CKD-MBD biomarker has not yet been determined.

Therefore, the aims of the present study were to evaluate the effect of each CKD-MBD biomarker, namely, serum calcium, phosphorus, PTH and ALP, on all-cause mortality, and to compare the performance of these biomarkers in predicting mortality in a Taiwanese PD population. To that end, we conducted a nationwide, population-based analysis using a database established by the Taiwan Renal Registry Data System (TWRDS).

## Subjects and Methods

This study was approved by the ethics committee of Taipei Medical University-Institutional Review Board (Number: 20141017) and was carried out in accordance with the Declaration of Helsinki of 1975, as revised in 2000. The requirement for written informed consent was waived by the Review Board.

The TWRDS was established in 1987 for the purpose of the accreditation and quality-monitoring of dialysis units. All dialysis units in Taiwan are obligated to provide the TWRDS with appropriate information on a quarterly basis to obtain reimbursement from the National Health Insurance. In 1996, data collection was computerized, and in 1997, additional information, including comorbidities, rehabilitation status, indices of dialysis adequacy and mineral and bone metabolism, and serologic markers of viral hepatitis were included. The data maintained by the TWRDS provide not only a robust foundation for comprehensive monitoring of the dialysis quality at a national level, but are also an important resource for population-based epidemiologic research^21–24^.

### Study population

The investigated population consisted of 12,966 patients on PD from all 117 PD units across Taiwan, who were registered in the TWRDS between 2005 and 2012. Among the total 12,966 patients in the registry data, 8,514 patients remained on PD, while 3,577 patients shifted to HD and 875 patients received kidney transplantation by the end of 2012. Of these three groups, patients who remained on PD were older; patients who shifted to HD included more diabetics, and had longer PD vintage, higher serum ALP concentration, and renal creatinine clearance; and patients who received a kidney transplant included more men and had higher serum albumin, calcium, phosphorus, and PTH concentrations (Table 1). Among the entire PD population in the registry data, patients with missing PTH values were excluded from analysis as logical imputation was not feasible (n = 850). Finally, 12,116 patients were included in the current statistical analysis. Patients who remained on PD were followed until mortality or the end of the study, while those who switched to HD or received kidney transplantation were censored.

**Table 1:** Demographic characteristics and time-averaged laboratory data of the entire PD population in Taiwan from 2005 to 2012.

	Total	Remained PD	Shifted to HD	Kidney Transplant	*p* value
Number	12966	8514	3577	875	—
Age (year)	53 ± 16	54 ± 16	52 ± 16	39 ± 13	<0.0001
Male (%)	6022(46)	3880(46)	1679(47)	463(53)	<0.0001
DM (%)	4743(37)	3156(37)	1494(42)	93(11)	<0.0001
PD Vintage (year)^†^	3.8 ± 2.6	3.8 ± 2.6	5.4 ± 2.3	3.3 ± 2.5	<0.0001
Time-averaged laboratory data					
Albumin (g/dL)	3.7 ± 0.4	3.6 ± 0.4	3.7 ± 0.4	3.9 ± 0.3	<0.0001
Hb (g/dL)	10.1 ± 1.2	10.2 ± 1.2	10.0 ± 1.1	10.0 ± 1.2	<0.0001
Ca (mg/dL)	9.2 ± 0.7	9.2 ± 0.7	9.3 ± 0.6	9.4 ± 0.7	<0.0001
P (mg/dL)	5.2 ± 1.1	5.1 ± 1.0	5.2 ± 1.0	5.6 ± 1.1	<0.0001
PTH (pg/mL)	271.6 ± 181.0	270.8 ± 184.7	266.2 ± 168.9	302.0 ± 192.2	<0.0001
ALP (IU/L)	126.7 ± 98.1	126.1 ± 100.7	130.7 ± 93.5	115.7 ± 90.3	0.0002
Kt/V^‡^	2.1 ± 0.32	2.1 ± 0.31	2.1 ± 0.32	2.1 ± 0.34	<0.0001
Renal CCr (mL/min)	5.0 ± 1.7	4.9 ± 1.8	5.3 ± 1.7	4.5 ± 1.4	<0.0001

PD, peritoneal dialysis; HD, hemodialysis; DM, diabetes mellitus; Hb, Hemoglobin; Ca, serum total calicium; P, serum phosphorus; PTH, parathyroid hormone; ALP, total alkaline phosphatase; Renal CCr, renal creatinine clearance.

^†^PD vintage of each patient was calculated from the start of 2005 to termination of PD or the end of 2012. ^‡^Weekly Kt/V, including renal CCr.

### Study outcome and predictors

The main outcome was all-cause mortality, which was defined as loss from registration in the TWRDS. This definition was based on the complete, nationwide coverage of renal replacement treatment expenditure provided by the National Health Insurance policy.

Serum concentrations for calcium, phosphorus, PTH, and ALP were analysed as potential predictors of mortality. The measuring and monitoring of these laboratory data were specified strictly by the TWRDS. Serum calcium and phosphorus levels were measured monthly and reported to the TWRDS every 3 months. Serum total calcium was corrected using the following formula: corrected calcium = (0.8 × [normal albumin level – exact albumin level]) + measured serum calcium. PTH was measured and reported to the TWRDS every 3 months. The majority of PTH tests across hospitals in Taiwan (97.9%) were performed by the Union Clinical Laboratory, where the second-generation PTH Chemiluminescence assay ADVIA Centaur (Siemens Healthcare Diagnostics, Tarrytown, NY) has been employed since 2007 (reference range, 14–72 pg/mL). Compared with the second-generation intact PTH Allegro Nichols IRMA suggested by the KDIGO guidelines in 2009, this commercial assay exhibited variation of 60–152%^25^. Total ALP was reported every 3 months as specified by the TWRDS, which was tested using the modified International Federation of Clinical Chemistry ADVIA 1800 (Siemens Healthcare Diagnostics, Tarrytown, NY).

### Statistical analysis

Descriptive statistics are expressed as mean ± standard deviation (SD) or as frequencies (percentages). Time-averaged values of biochemical and hematologic parameters were used for descriptive statistics. The time-averaged values were defined as the average values across the entire registry. Significance was evaluated using one-way analyses of variance (ANOVA) or chi-square tests, as appropriate.

A Cox proportional regression model and Kaplan-Meier method were performed with mortality as the outcome, censoring patients who shifted to HD or received a kidney transplant. For the Kaplan-Meier analyses, baseline data were used and the significance of survival differences was evaluated using the log-rank test. Multiple comparisons of Kaplan-Meier curves were conducted with Sidak correction. The baseline values were defined as the average value in the first year in the registry. To build the multivariate Cox regression model, all potential predictor variables were evaluated for association to mortality using the univariate Cox regression method. The serum concentrations of calcium, phosphorus, PTH, and ALP were considered as potential predictors. The pre-defined criteria for inclusion in the multivariate Cox regression model consisted of a univariate *p*-value < 0.2. In addition, all continuous variables were evaluated for collinearity by calculating the Pearson correlation coefficient. To reveal a potential non-linear association of predictors with mortality, a multivariate Cox regression model with the potential predictors represented as categorical variables was performed. Considering the U-shape association between PTH and mortality recently reported by Rhee *et al*.^20^ and the reference range provided in the KDIGO guidelines^14^, PTH values were categorized into 5 groups (<50, 50–150, 150–300, 300–600, and ≧600 pg/mL). Similarly, calcium values were categorized into 4 groups (<8.5, 8.5–9.5, 9.5–10.5, and ≧10.5 mg/dL) and phosphorus values were categorized into 6 groups (<3.5, 3.5–5.5, 5.5–6.5, 6.5–7.5, 7.5–8.5, and ≧8.5 mg/dL). In a large-scale study conducted in patients on PD by Rhee *et al*.^20^, the risk of mortality increased at an ALP level ≧150 IU/L. Therefore, ALP values were classified into 4 groups (<50, 50–100, 100–150, and ≧150 IU/L). As described previously, patients with missing PTH values were excluded from the study population. For missing values in descriptive statistics, the available data were used.

For survival analysis, patients with missing values for variables used in the regression model were excluded from the respective analysis. In addition to the Cox regression model using baseline data, time-dependent analyses were performed. In time-dependent analyses, calcium, phosphorus, PTH, and ALP were used as time-dependent covariates. Each time-dependent covariate was updated every 3 months. Missing values for time-dependent covariates were imputed by the last value carried forward method. However, patients with missing baseline values were excluded from time-dependent analyses as logical imputation was not feasible.

All statistical analyses were performed using SPSS, version 17.0. (SPSS Inc., Chicago, IL, USA) and SAS, version 9.1 (SAS Institute, Cary, NC, USA).

## Results

### Cohort description

For the 12,116 included patients, the mean age was 52 ± 16 years and the mean PD vintage was 3.9 ± 2.5 years. At the end of the 8-year study period, 3,036 (25%) patients were deceased. Among the included patients, higher PTH concentration was associated with younger age, and fewer male and diabetic patients. Mortality and PD vintage were significantly different among PTH groups. The PTH < 150 pg/mL group showed the highest mortality while the PTH 300–600 pg/mL group showed the longest PD vintage. The PTH ≧600 pg/mL group was associated with lower haemoglobin, higher phosphorus, higher ALP, and higher Kt/V. According to the KDIGO practice guidelines^26^, the haemoglobin level of patients in the PTH ≧600 pg/mL group was lower than normal, while the serum phosphorus level in all groups was higher than normal (>5.0 mg/dL)^14^. All groups had weekly Kt/V values that met the recommended dose (>1.7 per week) (Table 2).

**Table 2:** Demographic characteristics and time-averaged laboratory data of the Included PD patients.

	PTH (pg/mL)					*p* value
	Total	<150	150–300	300–600	≧600	
Number	12,116	3653	3829	3978	656	—
Age (year)	52 ± 16	57 ± 16	53 ± 15	48 ± 15	45 ± 15	<0.0001
Male (%)	5632(46)	1752(48)	1850(48)	1765(44)	265(40)	<0.0001
DM (%)	4435(37)	1785(49)	1487(39)	1025(26)	138(21)	<0.0001
PD vintage (year)	3.9 ± 2.5	3.4 ± 2.4	3.8 ± 2.4	4.4 ± 2.6	3.5 ± 2.7	<0.0001
Mortality (%)	3036(25)	1225(34)	942(25)	721(18)	148(23)	<0.0001
Time-averaged laboratory data						
Albumin (g/dL)	3.7 ± 0.4	3.7 ± 0.4	3.7 ± 0.4	3.8 ± 0.4	3.8 ± 0.4	<0.0001
Hb (g/dL)	10.1 ± 1.2	10.2 ± 1.2	10.2 ± 1.2	10.1 ± 1.1	9.9 ± 1.20	<0.0001
Ca (mg/dL)	9.3 ± 0.7	9.3 ± 0.6	9.2 ± 0.6	9.3 ± 0.7	9.3 ± 0.8	<0.0001
P (mg/dL)	5.2 ± 1.0	5.2 ± 1.0	5.1 ± 1.0	5.4 ± 1.0	5.8 ± 1.1	<0.0001
ALP (IU/L)	125.3 ± 94.1	125.3 ± 94.1	120.0 ± 89.6	132.5 ± 94.6	166.2 ± 120.0	<0.0001
Kt/V	2.07 ± 0.31	2.04 ± 0.33	2.06 ± 0.31	2.09 ± 0.31	2.10 ± 0.31	<0.0001
Renal CCr	5.0 ± 1.7	5.0 ± 1.7	5.0 ± 1.7	4.7 ± 1.5	4.7 ± 1.5	<0.0001

PD, peritoneal dialysis; DM, diabetes mellitus; Hb, Hemoglobin; Ca, serum total calicium; P, serum phosphorus; PTH, parathyroid hormone; ALP, total alkaline phosphatase; Renal CCr, renal creatinine clearance.

^†^PD vintage of each patient was calculated from the start of 2005 to termination of PD or the end of 2012. ^‡^Weekl Kt/V, including renal CCr.

### Survival analysis

Univariate Cox proportional regression analyses showed that age, sex, diabetic status, weekly Kt/V, renal creatinine clearance, albumin, and haemoglobin, along with our presumed predictors (calcium, phosphorus, PTH, and ALP), were significantly associated with mortality (*p* < 0.2, Supplementary Table 1). All continuous variables were found to be weakly correlated (0.2 < |r| ≦ 0.5) or uncorrelated (|r| ≦ 0.2) with each other and were included in the multivariate Cox regression model (Supplementary Table 2).

In the multivariate Cox regression model, increased age was associated with increased mortality while higher weekly Kt/V and albumin level were associated reduced mortality. Diabetes status and haemoglobin concentration were not associated with the hazard ratio of mortality. In contrast to the general expectation, higher renal creatinine clearance was associated with increased mortality. Of note, renal creatinine clearance was not included in the time-dependent analysis due to a large number of missing baseline values (n = 1,879).

**Figure 1:**
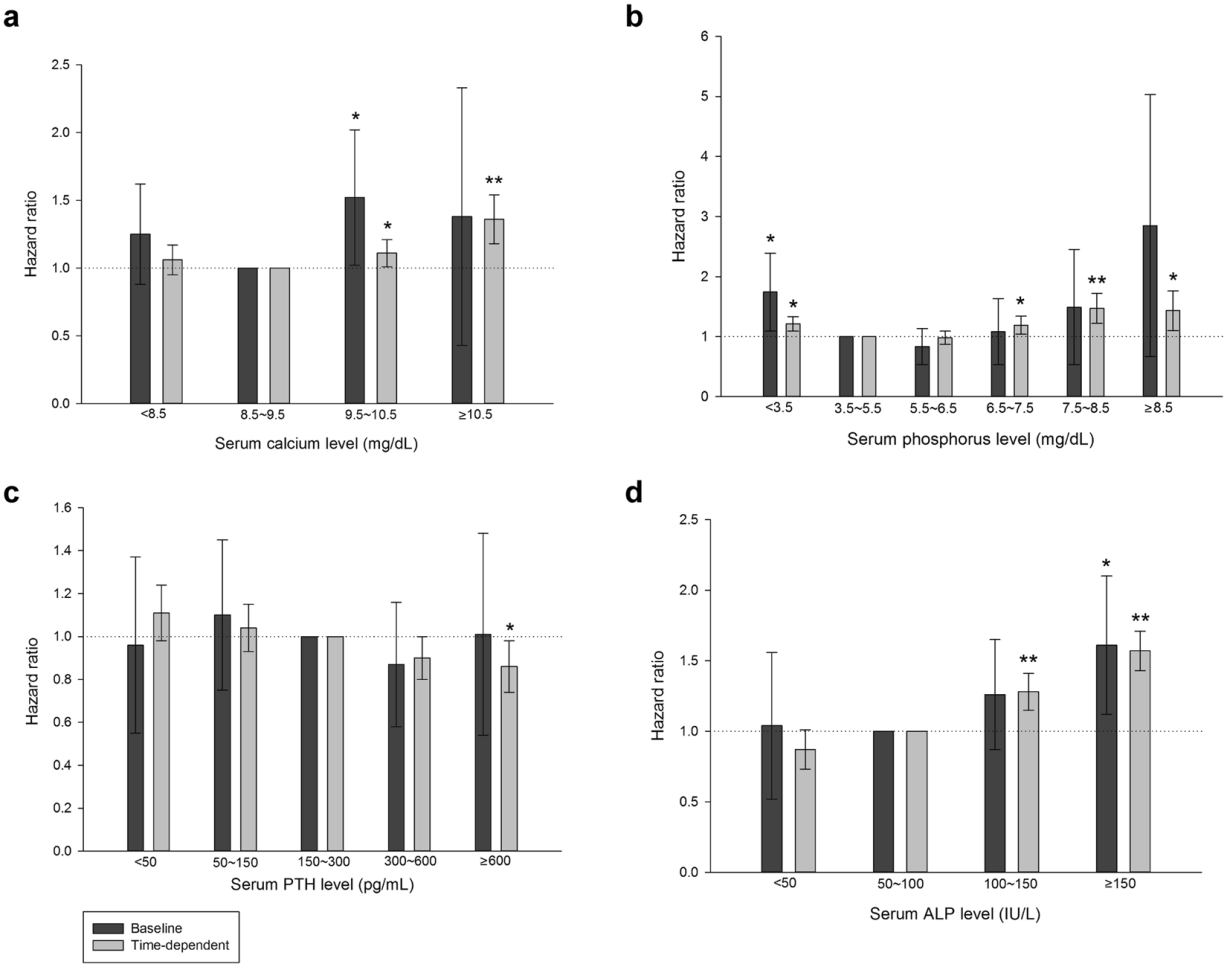
Cox proportional model showing the hazard ratios of CKD-MBD biomarkers to all-cause mortality in patients on peritoneal dialysis in Taiwan. Cox proportional regression model was adjusted for age, gender, diabetes mellitus, Kt/V, albumin, haemoglobin and renal creatinine clearance. Renal creatinine clearance was not included in the time-dependent analysis due to large number of missing baseline values (n = 1879). (**a**) Grouped by serum calcium levels (**b**) Grouped by serum phosphorus levels (**c**) Grouped by serum PTH levels (**d**) Grouped by serum total ALP levels. CKD-MBD, chronic kidney disease-mineral bone disorder; PTH, parathyroid hormone; ALP, total alkaline phosphatase. **p < 0.0001; *p < 0.05.

The majority of the findings were consistent whether using baseline data or time-dependent covariates; however, male sex was associated with increased mortality only in the time-dependent analysis (Table 3). Additionally, in the time-dependent analysis, serum calcium levels ≧9.5 mg/dL exhibited higher mortality. In contrast, in the analysis using baseline data, calcium levels either higher or lower than the normal range (8.5–10.5 mg/dL) were not consistently associated with increased mortality (Fig. 1a). Furthermore, in the time-dependent analysis, higher serum phosphorus was found to be positively associated with mortality at ≧7.5 mg/dL, while higher serum phosphorus levels were not associated with increased mortality in the analysis using baseline data. In contrast, serum phosphorus levels <3.5 mg/dL were consistently associated higher mortality in both baseline and time-dependent analyses (Fig. 1b).

**Table 3:** Multivariate Cox proportional hazard ratio of mortality in which PTH, ALP, calcium and phosphorus were considered as categorical variables.

	Baseline			Time-dependent		
	HR	95% CI	*p* value	HR	95% CI	*p* value
Age (per 10 year increments)	1.21	1.08~1.34	0.0007	1.13	1.10~1.16	<0.0001
Male	1.05	0.77~1.43	0.7498	1.11	1.02~1.20	0.0153
DM	1.06	0.78~1.45	0.6964	1.02	0.94~1.11	0.6073
Kt/V (per 1 increment)	0.52	0.33~0.83	0.0061	0.68	0.61~0.75	<0.0001
Albumin (per g/dL increment)	0.56	0.39~0.82	0.0028	0.31	0.29~0.34	<0.0001
Hb (per g/dL increment)	0.89	0.78~1.01	0.0809	1	0.98~1.03	0.8929
Ca (mg/dL)						
<8.5	1.25	0.88~1.79	0.2148	1.06	0.95~1.18	0.2962
8.5~9.5	Ref	—	—	Ref	—	—
9.5~10.5	1.52	1.02~2.27	0.0276	1.11	1.01~1.22	0.0353
≧10.5	1.38	0.43~4.44	0.8687	1.36	1.18~1.56	<0.0001
P (mg/dL)						
<3.5	1.74	1.09~2.27	0.0198	1.21	1.09~1.35	0.0005
3.5~5.5	Ref.	—	—	Ref	—	—
5.5~6.5	0.83	0.53~1.31	0.4333	0.98	0.87~1.09	0.6490
6.5~7.5	1.08	0.53~2.22	0.8261	1.19	1.04~1.37	0.0127
7.5~8.5	1.49	0.53~4.20	0.4461	1.47	1.22~1.77	<0.0001
≧8.5	2.85	0.67~12.09	0.1551	1.43	1.10~1.86	0.0079
PTH (pg/mL)						
<50	0.96	0.55~1.66	0.8834	1.11	0.98~1.24	0.0985
50~150	1.10	0.75~1.63	0.6225	1.04	0.93~1.16	0.4726
150~300	Ref.	—	—	Ref	—	—
300~600	0.87	0.58~1.31	0.5130	0.90	0.80~1.01	0.0806
≧600	1.01	0.54~1.89	0.9832	0.86	0.74~0.99	0.0377
ALP (IU/L)						
<50	1.04	0.52~2.09	0.9216	0.87	0.73~1.03	0.1072
50~100	Ref	—	—	Ref	—	—
100~150	1.26	0.87~1.83	0.2185	1.28	1.15~1.41	<0.0001
≧150	1.61	1.12~2.31	0.0100	1.57	1.43~1.72	<0.0001
Renal CCr (per mL/min increment)^†^	1.13	1.06~1.19	<0.0001	—	—	—

PTH, parathyroid hormone; ALP, total alkaline phosphatase; HR, hazard ratio; CI, confidence interval; DM, diabetes mellitus; Kt/V, weekly Kt/V; Hb, hemoglobin, Ca, total calcium; P, phosphorus; renal CCr, renal creatinine clearance.

^†^Renal creatinine clearance was not included in the time-dependent analysis due to large number of missing baseline values (n = 1879).

In the time-dependent analysis, PTH levels ≧600 pg/mL were associated with lower mortality compared to reference range (150–600 pg/mL), while PTH levels lower than reference range (<150 pg/mL) showed an insignificant trend of increasing mortality. In the analysis using baseline data, PTH levels either lower or higher than the reference range were not associated with higher mortality (Fig. 1c). In the time-dependent analysis, serum ALP levels ≧100 IU/L were associated with increased mortality. In the analysis using baseline data, higher ALP levels was associated higher mortality at ≧150 IU/L. In both analyses, lower ALP levels (<50 IU/L) were not associated with mortality (Fig. 1d).

**Figure 2:**
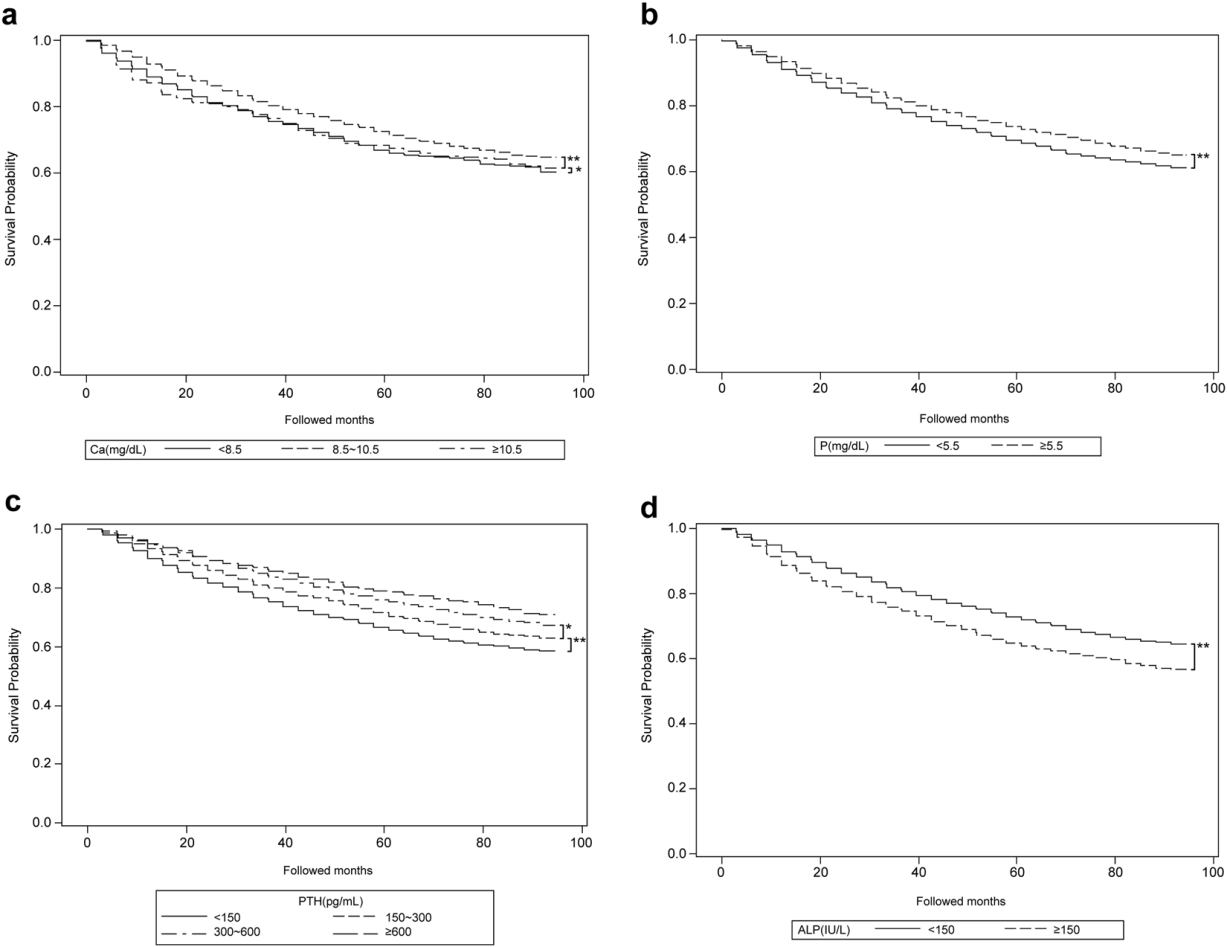
Kaplan-Meier curves showing the survival of peritoneal dialysis patients in Taiwan categorized by CKD-MBD biomarkers. Significance was tested by log-rank test. (**a**) Grouped by serum calcium (**b**) Grouped by serum phosphorus (**c**) Grouped by serum PTH (**d**) Grouped by serum total ALP. CKD-MBD, chronic kidney disease-mineral bone disorder; PTH, parathyroid hormone; ALP, alkaline phosphatase. Multiple comparison was performed by Sidak correction. **p < 0.0001; *p < 0.05.

In the Kaplan-Meier analyses, serum calcium levels of 8.5–10.5 mg/dL showed superior survival probability compared to that for either higher or lower levels. The survival probabilities of the three groups stratified by serum calcium levels were significantly different by a Log-rank test (*p* < 0.0001, Fig. 2a). The survival probabilities among calcium levels significantly differed from each other (Table 4). Phosphorus levels ≧5.5 mg/dL showed a significantly higher survival probability compared to that for levels <5.5 mg/dL, contrary to our findings in the Cox regression model (*p* < 0.0001, Fig. 2b). PTH levels ≧600 pg/mL showed the highest survival and PTH levels <150 pg/mL showed the lowest survival. The survival was significantly different among PTH groups (*p* < 0.0001, Fig. 2c). The survival for PTH levels <150 pg/mL was significantly lower compared to that for all other PTH levels, while the survival for PTH levels ≧600 pg/mL was not significantly higher compared to that for PTH levels 150–300 or 300–600 pg/mL. The survival for PTH levels 300–600 pg/mL was significantly higher compared to that for PTH levels 150–300 pg/mL (Table 4). ALP levels <150 U/L showed a significantly higher survival probability compared to that for levels ≧150 U/L (*p* < 0.0001, Fig. 2d).

**Table 4:** Multiple comparisons of Kaplan-Meier curves conducted by Sidak correction.

Serum calcium concentrations (mg/dL)			*p* value
<8.5	To	8.5~10.5	<0.0001
<8.5		≧10.5	0.0134
8.5~10.5		≧10.5	<0.0001
Serum PTH concentrations (pg/mL)			
<150	To	150~300	<0.0001
<150		300~600	<0.0001
<150		≧600	<0.0001
150~300		300~600	0.0021
150~300		≧600	0.1231
300~600		≧600	0.1223

PTH, parathyroid hormone.

## Discussion

In the present study, the Kaplan-Meier curves and Cox proportional models using baseline data and time-dependent covariates demonstrated inconsistent effects of serum calcium and phosphorus on mortality in PD patients. However, since models using time-varying explanatory variables are in general more robust, several trends can be summarized from our findings as follows: 1) A higher serum calcium level was associated with increased mortality. 2) Serum phosphorus level ≧6.5 mg/dL or <3.5 mg/dL were associated with increased mortality. 3) A higher serum PTH level was not associated with increased mortality. 4) A higher serum ALP level was associated with increased mortality with a cut-off value of 100–150 IU/L.

In the present study, the association between CKD-MBD biomarkers and all-cause mortality was inconsistent between statistical models using baseline measurements or time-varying covariates. This suggests that in a cohort study with a long follow-up period, the use of baseline measurements as predictors in statistical models overlooks important fluctuations in the predictor variables across the study period. Accordingly, in a registration-based study with repeated measurements, time-dependent analyses theoretically provide a more appropriate evaluation of the association between the time-varying covariates and outcomes.

The majority of observational studies conducted in HD patients have shown that serum phosphorus and calcium levels either higher or lower than the normal limits are associated with a higher risk of mortality, depicting U-shaped relationships^27–31^. Noordij *et al*.^17^, in a prospective cohort study involving 586 PD patients, demonstrated that serum phosphorus levels higher than the normal upper limit, but not abnormal calcium or PTH levels, were associated with increased mortality. Similarly, in another prospective cohort study involving 515 dialysis patients (including 158 PD patients) conducted by Stevens *et al*.^16^, only serum phosphorus (not calcium or PTH) showed significant association with mortality. In the present study, conducted exclusively in PD patients, time-dependent analyses showed that serum calcium levels ≧9.5 mg/dL, but not hypocalcemia, was significantly associated with increased mortality. This suggests a deleterious effect of higher serum calcium levels on the survival of patients on PD. In addition, the results of the present study showed that the effect of serum phosphorus on mortality depicted a U-shaped relationship. While the time-dependent analysis showed increased mortality with serum phosphorus levels ≧6.5 mg/dL, phosphorus levels <3.5 mg/dL were consistently associated with increased mortality in statistical models using either baseline or time-varying variables. This may suggest that malnutrition has a strong detrimental effect in patients on PD, regardless of its duration.

The relationship between PTH and mortality in dialysis patients has also been inconsistent across multiple studies. While some studies have shown that higher PTH is associated with decreased risk of mortality^3–7, 9^, others have conversely suggested an increased risk of mortality^10, 32^. In addition, several studies have shown a U-shaped relationship^5, 27–29, 31^ or no significant association^1, 2, 8, 33^ between PTH and mortality. In a prospective study, which included 277 PD patients, Avram *et al*.^32^ demonstrated that lower PTH is associated with increased mortality. However, they considered PTH as a continuous variable; thus, a non-linear association may have been overlooked. More recently, Rhee *et al*.^20^ conducted a retrospective cohort study enrolling 9,244 patients on PD which evaluated the impact of PTH and ALP on mortality. In their study, PTH had a U-shaped association with mortality, with a concentration of 200–700 pg/mL exhibiting the lowest mortality and a concentration <100 pg/mL exhibiting the highest mortality. Partly in line with these results, the present study showed that a higher PTH level was not associated with increased mortality in PD patients. However, a U-shaped association of PTH to mortality in patients on PD was not demonstrated in the present study.

Recently, evidence that the serum ALP levels are associated with adverse outcomes for dialysis patients, including cardiac failure^11, 12^, renal osteodystrophy^34–36^, and mortality has been accumulating^20, 37, 38^. These findings suggest that ALP might be a valuable parameter in the treatment of CKD-MBD in dialysis patients. In Rhee *et al*.^20^, higher ALP in patients on PD was associated with increased mortality in a binary fashion, with a cut-off value of ≧150 IU/L. The results of the present study showed a similar trend, supporting the role of ALP as a predictor of mortality in patients on PD.

Considering the results for PTH and ALP, our study revealed similar findings to the work of Rhee *et al*.^20^, which was another large-scaled, retrospective cohort study in patients on PD. However, the race and ethnicity of the subjects in this previous study consisted mainly of white, black, and Hispanic. In contrast, the present study is comprised of exclusively Asian people, and therefore, the present study extends the generalisation of these findings to a wider racial spectrum.

The multivariate Cox regression model paradoxically demonstrated that renal creatinine clearance was associated with increased mortality. One explanation to this phenomenon is that this study started with a cross-sectional cohort; thus, the status and duration of dialysis before the onset of the registry was not available. Possibly, the patients with lower renal creatinine clearance represented those who had longer dialysis vintage and who were more stable patients. This may have led to a bias. Another explanation is that, as reported recently, urinary creatinine clearance is prone to falsely overestimate glomerular filtration rate in patients with renal impairment and is less reliable in patients with end-stage kidney disease^39^. In addition, since renal creatinine clearance was calculated as the filtered creatinine per minute divided by serum creatinine concentration, patients with malnutrition and lower muscle mass may have exhibited lower serum creatinine levels and therefore, higher renal creatinine clearance. This may also be an explanation for the unexpected finding in the present study.

There were a number of limitations in the present study. One limitation is that the study started with a cross-sectional cohort; therefore, patients who switched from HD to PD before the beginning of the registry were also included, which may have affected the results. The outcome of the current study, all-cause mortality, was defined as loss from registration in TWRDS. However, this definition was not cross-referenced with national vital statistics and might over-estimate the actual mortality. Other limitations include that only total ALP, not bone-specific ALP was available, and that the PTH assay used was a second generation assay with high cross-reactivity to inactive PTH fragments. In addition, the actual medical records of each individual were not available; therefore, information about cardiovascular diseases and other comorbidities, medication, dialysate calcium concentration, and the cause of mortality were absent from our data. However, the main strengths of this study include the use of a large, population-based database with nationwide coverage of PD patients in Taiwan with laboratory data that was strictly specified and measured, and the use of time-dependent variables in the statistical analyses.

It has been argued that features of CKD-MBD differ between patients undergoing PD and HD in that a dynamic bone disease was more frequent, and bone microarchitecture was less affected, in patients on PD^40, 41^; nevertheless, studies comparing the effects of CKD-MBD biomarkers to mortality in patients on PD and HD remain limited. Currently, the KDIGO does not recommend specific guidelines on the management of CKD-MBD for patients on PD or HD. A recent nationwide, registry-based study conducted with patients on HD in Taiwan found the following: 1) higher ALP level was associated with increased mortality in a binary fashion, with a cut-off value of ≧100 IU/L; 2) a PTH level <100 pg/mL was associated increased mortality, while higher PTH levels not being associated with increased mortality; and 3) both serum calcium and phosphorus were associated with mortality in a U-shaped fashion, with the nadir located at 9.5–10.5 mg/dL and 5.5–6.5 mg/dL, respectively^42^. The U-shaped associations for serum calcium and phosphorus with regard to mortality reported in the work by Lin *et al*., are in line with most observational studies conducted with patients on HD^27–31^. While an association between serum phosphorus and mortality has been inconsistently reported across multiple studies conducted with patients on PD, several studies have reported no effects of serum calcium and PTH on mortality^16, 17^. In another large-scaled retrospective cohort study conducted with HD patients in the U.S.A., higher ALP level was associated with increased mortality in a binary fashion with a cut-off at ≧120 IU/L^43^. Comparing these HD-related findings to the present study, it is possible that in the PD population, serum calcium and phosphorus may have a less prominent effect on mortality, while ALP has a more consistent effect on mortality. In addition, several recent studies have shown that in both patients on HD and PD, higher PTH levels did not increase mortality. However, the evidence to date remains insufficient to provide recommendations specifically for PD patients. We expect that further research on CKD-MBD in PD patients could contribute to a practice guideline being developed specifically for this population in the future.

## Electronic supplementary material

supplement file
