# Supplementary material for: Roles of Serum Calcium, Phosphorus, PTH and ALP on Mortality in Peritoneal Dialysis Patients: A Nationwide, Population-based Longitudinal Study Using TWRDS 2005–2012
Source: Sci Rep. 2017 Feb 24;7:33. doi: 10.1038/s41598-017-00080-4 (PMC5428378; doi:10.1038/s41598-017-00080-4)
Supplement: Supplementary file 1 — supplement file [file 41598_2017_80_MOESM1_ESM.doc]

**Roles of Serum Calcium, Phosphorus, PTH and ALP on Mortality in Peritoneal Dialysis Patients: a Nationwide, Population-based Longitudinal Study Using TWRDS 2005-2012**

Authors:

Chung-Te Liu^1,3,4^, Yen-Chung Lin^2,3,4*^, Yi-Chun Lin^5,6^, Chih-Chin Kao^2,3,4^, Hsi-Hsien Chen^2,3^, Chih-Cheng Hsu^7^, Mai-Szu Wu^2,3*^

Affiliations:

^1^Division of Nephrology, Department of Internal Medicine, Taipei Medical University-Wanfang Hospital

^2^Division of Nephrology, Department of Internal Medicine, Taipei Medical University Hospital

^3^Department of Internal Medicine, School of Medicine, College of Medicine, Taipei Medical University, Taipei, Taiwan;

^4^Graduate Institute of Clinical Medicine, College of Medicine, Taipei Medical University, Taipei, Taiwan

^5^Division of Endocrinology & Metabolism, Department of Medicine, Taipei Veterans General Hospital, Taipei, Taiwan;

^6^Faculty of Medicine, National Yang-Ming University, Taipei, Taiwan

^7^ Institute of Population Health Sciences, National Health Research Institutes, Zhunan, Taiwan.

*Corresponding authors:

Email address: maiszuwu@gmail.com (MW) and yclin0229@tmu.edu.tw (YL)

Yen-Chung Lin and Mai-Szu Wu contributed equally to this work.

| Supplementary Table 1. Univariate Cox proportional hazard ratio of mortality. | | | |
| --- | --- | --- | --- |
|  | HR | 95% CI | *p* value |
| Age (per 10 years increase) | 1.33 | 1.30~1.36 | <0.0001 |
| Male | 1.13 | 1.06~1.21 | 0.0002 |
| DM | 1.56 | 1.46~1.67 | <0.0001 |
| Kt/V (per 1 increase) | 0.47 | 0.41~0.53 | <0.0001 |
| Albumin (per 1 g/dL increase) | 0.27 | 0.25~0.29 | <0.0001 |
| Hb (per 1 g/dL increase) | 0.93 | 0.90~0.96 | <0.0001 |
| Ca (per 1 mg/dL increase) | 0.68 | 0.64~0.72 | <0.0001 |
| P (per 1 mg/dL increase) | 0.78 | 0.75~0.81 | <0.0001 |
| ALP (per 50 IU/L increase) | 1.06 | 1.04~1.07 | <0.0001 |
| PTH (per 50 pg/mL increase) | 0.92 | 0.91~0.93 | <0.0001 |
| Renal CCr (per mL/min increase) | 1.29 | 1.26~1.32 | <0.0001 |

HR, hazard ratio; CI, confidence interval; DM, diabetes mellitus; Kt/V, weekly Kt/V, including renal creatinine clearance; Hb, hemoglobin; Ca, calcium; P, phosphorus; ALP, total alkaline phosphatase; PTH, parathyroid hormone; renal CCr, renal creatinine clearance.

Supplementary Table 2. Linear correlation between continuous variables.

| R  Sig.  N | Age | Alb | Hb | Ca | P | ALP | PTH | Kt/V | CCr |
| --- | --- | --- | --- | --- | --- | --- | --- | --- | --- |
| Age | 1  -  12966 | -0.3852  <0.0001  12484 | 0.0703  <0.0001  12467 | -0.0936  <0.0001  12077 | -0.3703  <0.0001  12459 | 0.0119  0.1864  12340 | -0.2444  <0.0001  12116 | -0.0663  <0.0001  11756 | 0.2354  <0.0001  9381 |
| Alb |  | 1  -  12484 | 0.1593  <0.0001  12447 | 0.3369  <0.0001  12064 | 0.3192  <0.0001  12446 | -0.0954  <0.0001  12335 | 0.1924  <0.0001  12110 | 0.1341  <0.0001  11743 | -0.3362  <0.0001  9375 |
| Hb |  |  | 1  -  12467 | 0.0380  <0.0001  12068 | -0.1560  <0.0001  12443 | -0.0253  0.0049  12326 | -0.0586  <0.0001  12107 | 0.0319  0.0005  11727 | 0.1026  <0.0001  9378 |
| Ca |  |  |  | 1  -  12077 | 0.1177  <0.0001  12068 | 0.0039  0.6701  11953 | 0.0107  0.2481  11746 | 0.1421  <0.0001  11372 | -0.1625  <0.0001  9121 |
| P |  |  |  |  | 1  -  12459 | -0.0502  <0.0001  12336 | 0.3133  <0.0001  12112 | -0.1380  <0.0001  11733 | -0.4038  <0.0001  9378 |
| ALP |  |  |  |  |  | 1  -  12340 | 0.1238  <0.0001  12067 | -0.0597  <0.0001  11682 | 0.1465  <0.0001  9319 |
| PTH |  |  |  |  |  |  | 1  -  12116 | 0.0566  <0.0001  11554 | -0.1418  <0.0001  9258 |
| Kt/V |  |  |  |  |  |  |  | 1  -  11756 | 0.0031  0.7658  9002 |
| CCr |  |  |  |  |  |  |  |  | 1  -  9381 |

R, Pearson correlation coefficient; Sig, significance; N, number; Alb, albumin; Hb, hemoglobin; Ca, serum total calcium, P, serum phosphorus; ALP, serum total alkaline phosphatase; PTH, serum parathyroid hormone; Kt/V, weekly Kt/C, including renal creatinine clearance; CCr, renal creatinine clearance.
